# Supplementary material for: Phage-microbe dynamics after sterile faecal filtrate transplantation in individuals with metabolic syndrome: a double-blind, randomised, placebo-controlled clinical trial assessing efficacy and safety
Source: Nat Commun. 2023 Sep 12;14:5600. doi: 10.1038/s41467-023-41329-z (PMC10497675; doi:10.1038/s41467-023-41329-z)

Phage-microbe dynamics after sterile faecal filtrate transplantation in individuals with metabolic syndrome: a double blind, randomised, placebo-controlled clinical trial assessing efficacy and safety

Koen Wortelboer, Patrick A. de Jonge, Torsten P.M. Scheithauer, Ilias Attaye, E. Marleen Kemper, Max Nieuwdorp, Hilde Herrema

Overview of supplementary information

Supplementary Figure 1. Overview of recruitment and screening.

Supplementary Figure 2. Beta diversity in the phageome.

Supplementary Figure 3. Faecal filtrate production and administration, and sequencing pipeline.

Supplementary Table 1. Baseline dietary intake of participants.

Supplementary Table 2. Results from the continuous glucose monitoring devices.

Supplementary Table 3. In- and exclusion criteria for study participants.

Supplementary Table 4. In- and exclusion criteria for faeces donors.

CONSORT Flow Diagram

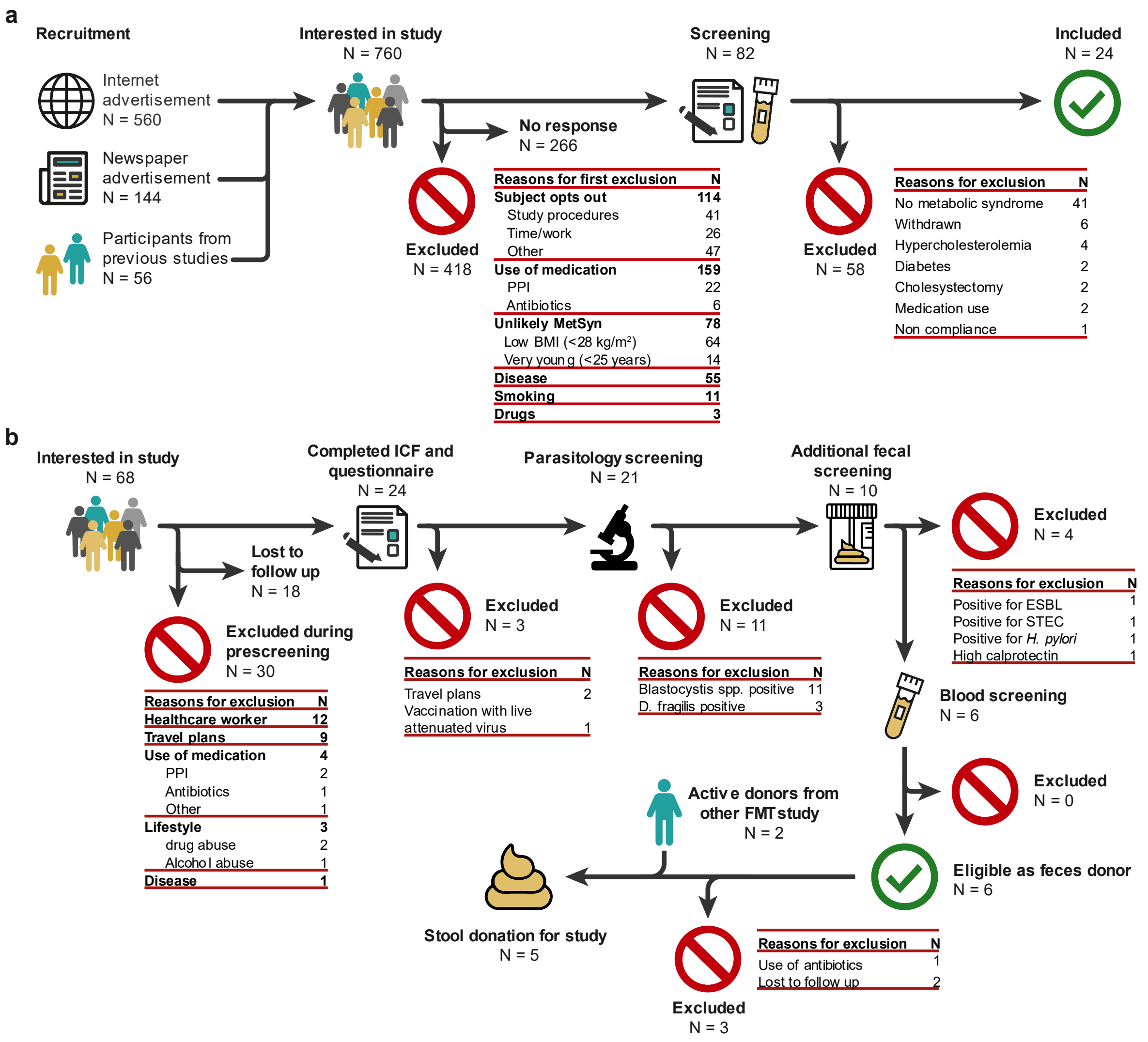

Supplementary Figure 1. Overview of recruitment and screening. a Recruitment and screening of participants with the metabolic syndrome. **b** Recruitment and screening of healthy donors for the sterile faecal filtrate. BMI = Body Mass Index; D. fragilis = Dientamoeba fragilis; ESBL = extended spectrum beta-lactamase producer FMT = faecal microbiota transplantation; H. pylori = Helicobacter pylori; ICF = informed consent form; PPI = proton pump inhibitor; STEC = shigatoxigenic Escherichia coli.

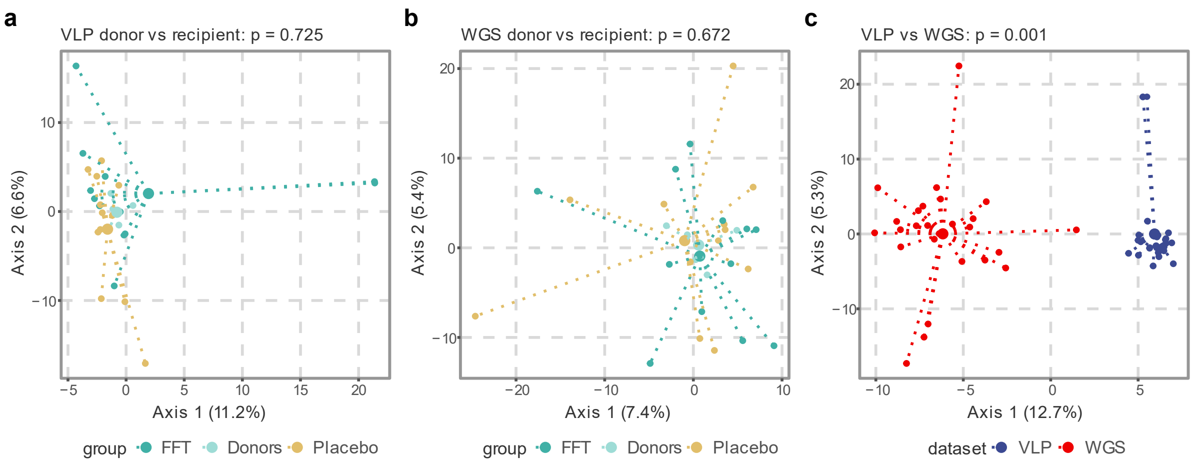

Supplementary Figure 2. Beta diversity in the phageome. a Principal component analysis (PCA) of the viral populations (VP) within the phage virions (VLP) and **b** bulk-derived phageome (WGS) between the subjects with metabolic syndrome in the faecal filtrate (FFT) and placebo groups and the healthy subjects who donated their stool at baseline. There were no differences in overall composition of the (pro)phages between the groups as determined by permutational analysis of variance (PERMANOVA). **c** PCA of the VPs showing the difference in overall composition between the free phages (VLP) and bulk-derived phageome (WGS), which was statistically significant as determined by PERMANOVA (p = 0.001).

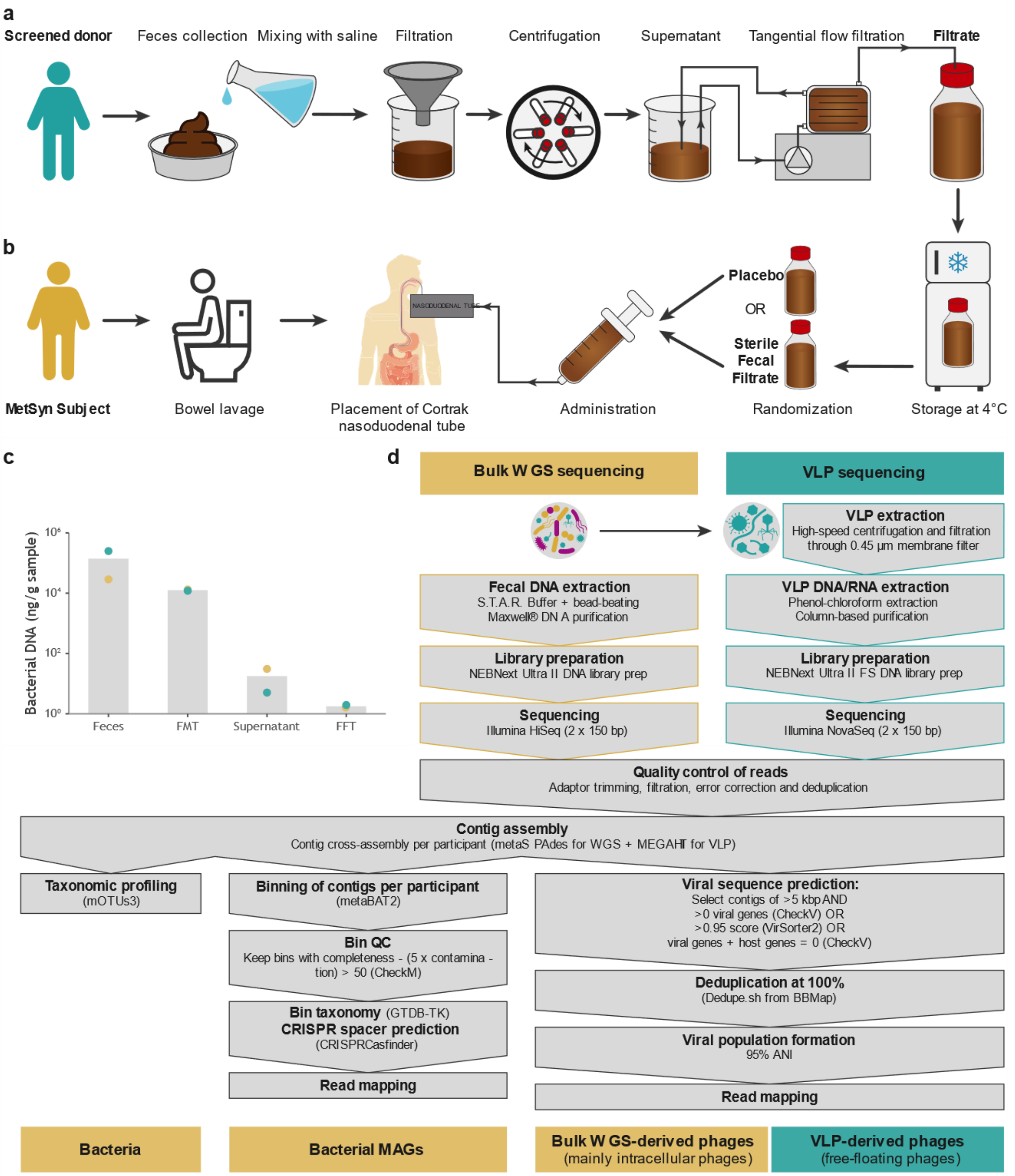

Supplementary Figure 3. Faecal filtrate production and administration, and sequencing pipeline. **a** Production of the sterile faecal filtrate. **b** Administration of the sterile faecal filtrate. **c** qPCR for the bacterial 16S rRNA gene showing a 10^5-fold decrease in bacterial DNA. **d** Overview of the used pipeline for the microbiome shotgun sequencing and the VLP shotgun sequencing. MAGs = Metagenome-assembled genomes; QC = quality control; VLP = viral-like particle; WGS = whole genome shotgun.

Supplementary Table 1. Baseline dietary intake of participants.

	Placebo (N = 12)		Fecal Filtrate (N = 12)		*p-value*
**Energy** (kcal)	**2199**	(271)	**2146**	(580)	0.44
**Fats** (g)	**93.4**	(18.9)	**93.3**	(29.7)	1.00
**Saturated fats** (g)	**33.8**	(9.5)	**33.0**	(11.8)	1.00
**Carbohydrates** (g)	**218.3**	(51.8)	**218.4**	(70.3)	0.98
**Sugars** (g)	**82.6**	(30.4)	**91.2**	(44.5)	0.71
**Proteins** (g)	**106.0**	(26.4)	**89.8**	(25.0)	0.16
**Fibers** (g)	**22.1**	(6.7)	**18.7**	(6.0)	0.10
**Salt** (g)	**7.72**	(2.93)	**7.50**	(2.86)	0.89

Data are reported as mean (SD). Statistical testing between the placebo and fecal filtrate groups is performed by a two-sided independent Mann-Whitney U test. Source data are provided in the Source Data file.

Supplementary Table 2. Results from the continuous glucose monitoring devices.

		**Placebo** (N=12)	**Fecal Filtrate** (N=12)	***p-value***
**Mean glucose** (mmol/L)	Before	**5.36** (0.41)	**5.37** (0.70)	*0.75*
	After	**5.34** (0.38)	**5.33** (0.59)	
**SD glucose** (mmol/L)	Before	**0.89** (0.19)	**0.98** (0.42)	*0.58*
	After	**0.94** (0.26)	**0.94** (0.37)	
**CV glucose** (%)	Before	**16.7** (3.6)	**17.8** (5.1)	*0.39*
	After	**17.5** (4.7)	**17.3** (4.8)	
**Min glucose** (mmol/L)	Before	**3.55** (0.47)	**3.43** (0.37)	*0.37*
	After	**3.51** (0.55)	**3.46** (0.48)	
**Max glucose** (mmol/L)	Before	**8.63** (1.21)	**8.92** (1.74)	*0.33*
	After	**9.18** (1.44)	**8.82** (1.75)	
**Time between** 3.9-10 (%)	Before	**97.1** (4.5)	**95.5** (5.4)	*0.19*
	After	**97.3** (4.4)	**97.5** (3.3)	
	p-value	*1.00*	*0.01*	
**Est. HbA1c** (mmol/mol)	Before	**31.0** (2.8)	**31.1** (4.8)	*0.76*
	After	**31.0** (2.6)	**30.9** (4.1)	
**AUC/day**	Before	**7621** (991)	**7617** (823)	*0.55*
	After	**7795** (671)	**7841** (919)	
**AUC>2SD/day**	Before	**33.0** (23.5)	**32.3** (9.5)	*0.53*
	After	**41.4** (15.5)	**40.6** (13.9)	
**CONGA 1** score	Before	**0.99** (0.22)	**1.09** (0.42)	*0.28*
	After	**0.99** (0.25)	**1.08** (0.46)	
**MODD** score	Before	**0.82** (0.17)	**0.87** (0.30)	*0.41*
	After	**0.83** (0.20)	**0.92** (0.39)	
**MAGE** score	Before	**1.68** (0.87)	**1.81** (1.03)	*0.32*
	After	**1.45** (0.47)	**1.53** (0.72)	

Unless otherwise specified data are reported as mean (SD). Mixed model analyses were used to assess differences between groups and timepoints, whereafter post hoc analyses were performed with Bonferroni correction. All tests were two-sided. The p-values in the right column shows the overall effect of treatment on the variable and only when significant, the adjusted p-values from the post hoc tests are shown. The p-values underneath variables indicate statistically significant differences between before and after intervention within a treatment group. BMI = Body Mass Index; WHR = waist-hip ratio; BP = blood pressure; HOMA-IR = Homeostatic Model Assessment for Insulin Resistance; HDL = high-density lipoprotein; LDL = low-density lipoprotein; CRP = C-reactive protein. Source data are provided in the Source Data file.

Supplementary Table 3. In- and exclusion criteria for study participants.

INCLUSION CRITERIA

- Caucasian male or female
- Age: 18 - 65 years old
- BMI ≥ 25 kg/m^2
- At least 3 of the following criteria:
    - Fasting plasma glucose ≥ 5.6 mmol/L, or HOMA-IR index ≥2.5 (HOMA-IR is measured as (fasting insulin (pmol/L) x fasting glucose (mmol/L)) / 135)
    - Waist-circumference ≥ 102 cm for males, ≥ 89 cm for females
    - HDL-cholesterol ≤ 1.02 mmol/L for males, ≤ 1.29 mmol/L for females
    - Blood pressure ≥ 130/85 mmHg
    - Triglycerides ≥ 1.7 mmol/L
- Subjects should be able to give informed consent

EXCLUSION CRITERIA

- A history of cardiovascular event (e.g., CVA or MI) or pacemaker implantation
- Use of any medication including proton pump inhibitors, antibiotics, and pro-/prebiotics in the past three months or during the study period
- (Expected) prolonged compromised immunity (due to recent cytotoxic chemotherapy or HIV infection with a CD4 count < 240/mm3)
- Presence of overt T1DM or T2D
- History of chronic diarrhoea (≥3 stools/day for >4 weeks), chronic obstipation (<2 defecations/week for >3 months), IBS (according to Rome IV criteria), or IBD.
- Smoking or illicit drug use in the past three months or use during the study period
- Alcohol abuse (>5 units/day on average) in the past three months or use of > 2 units/day of alcohol during the study period
- History of cholecystectomy

Abbreviations: CVA = cerebrovascular accident; HDL = high-density lipoprotein; HIV = human immunodeficiency viruses; HOMA-IR = Homeostatic Model Assessment for Insulin Resistance; IBD = inflammatory bowel disease; IBS = irritable bowel syndrome; MI = myocardial infarction; T1DM = type 1 diabetes mellitus; T2DM = type 2 diabetes mellitus.

Supplementary Table 4. In- and exclusion criteria for faeces donors.

INCLUSION CRITERIA

- Caucasian male or female
- Age: 18 – 65 years old
- BMI: 18-25 kg/m^2
- Subjects should be able to give informed consent

EXCLUSION CRITERIA

Positive test for infectious agent

- Positive Dual Faeces Test for *Giardia Lamblia*, *Dientamoeba fragilis*, *Entamoeba histolytica*, *Microsporidium* spp., *Cryptosporidium* spp., *Cyclospora*, *Isospora,* or *Blastocystis Hominis*. Positive microscopic exam for eggs, cysts, and larvae (e.g. helminth eggs)
- Presence of faecal bacterial pathogens *Salmonella* spp., *Shigella* spp., *Campylobacter* spp., *Yersinia* spp., *C. difficile*, *H. pylori*, STEC, *Aeromonas* spp., or *Pleisiomonas shigelloides* in faeces
- Presence of ESBL producers, CRE, VRE, or MRSA in faeces
- Presence of *Rotavirus*, *Norovirus* I/II, *Enterovirus*, *Parechovirus*, *Astrovirus*, *Sapovirus,* or *Adenovirus* in faeces
- Presence of SARS-CoV-2 in faeces
- Positive serologic test for HIV 1/2, HAV, HBV, HCV, HEV, active CMV or EBV, *Strongyloides,* or *Treponema pallidum*

Risk of infectious agent

- History of, or known exposure to HIV, HBV, HCV, syphilis, HTLV I and II, malaria, trypanosomiasis, or tuberculosis
- Known systemic infection not controlled at the time of donation
- Unsafe sex practice
- Previous reception of tissue/organ transplant
- Previous (<12 months) reception of blood products
- Recent (<6 months) needle stick accident
- Recent (<6 months) body tattoo, piercing, earring, or acupuncture
- Recent medical treatment in poorly hygienic conditions
- Risk of transmission of diseases caused by prions
- Recent parasitosis or infection from rotavirus, Giardia lamblia, and other microbes with GI involvement
- Recent travel to tropical countries, countries at high risk of communicable diseases, or traveller's diarrhoea
- Recent (<6 months) history of vaccination with a live attenuated virus, if there is a possible risk of transmission
- Healthcare providers having frequent patient contact
- Individual working with animals

Gastrointestinal comorbidities

- History of IBS (according to Rome IV criteria), IBD, functional chronic constipation, or other chronic GI disorders
- History of chronic, systemic autoimmune disorders with GI involvement, such as coeliac disease
- History of, or high risk for, GI cancer, or polyposis
- Recent appearance of diarrhoea (≥3 stools/day), and/or haematochezia
- Elevated faecal calprotectin (> 50 µg/g)

Factors affecting intestinal microbiota composition

- Use of any medication including proton pump inhibitors, antibiotics, and pro-/prebiotics in the past three months or during the study period
- Smoking or illicit drug use in the past three months or during the study period
- History of cholecystectomy

Other conditions

- History of neurological or neurodegenerative disorders
- History of psychiatric conditions
- Presence of chronic low-grade inflammation or metabolic syndrome (NCEP criteria)
- Presence of T1DM, T2DM, or hypertension
- Alcohol abuse (>5 units/day on average) in the past three months or use of > 2 units/day of alcohol during the study period
- Abnormal liver or renal function (creatinine >110 µmol/l, ureum >8,2 mmol/l, ASAT > 40 U/L, ALAT > 45 U/L, AF > 120 U/L, GGT > 60 U/L, bilirubin >17µmol/L), or impaired immunity (CRP > 5 mg/L, haemoglobin < 8,5 mmol/L, MCV: 80-100 fL, leukocytes: 4,0- 10,5 x109/L, thrombocytes: 150-400 x10^9/L).

Abbreviations: AF = alkaline phosphatase; ALAT = alanine aminotransferase; ASAT = aspartate aminotransferase; CMV = cytomegalovirus; CRE = Carbapenem-resistant Enterobacteriaceae; CRP = C-reactive protein; EBV = Epstein–Barr virus; ESBL = extended spectrum beta-lactamase; GGT = gamma-glutamyl transferase; GI = gastrointestinal; HAV = hepatitis A virus; HBV = hepatitis B virus; HCV = hepatitis C virus; HEV = hepatitis E virus; HIV = human immunodeficiency viruses; HTLV = human T-lymphotropic virus; IBD = inflammatory bowel disease; IBS = irritable bowel syndrome; MCV = mean corpuscular volume; MRSA = methicillin-resistant Staphylococcus aureus; NCEP = National Cholesterol Education Program; SARS-CoV-2 = severe acute respiratory syndrome coronavirus 2; STEC = shigatoxigenic Escherichia coli; T1DM = type 1 diabetes mellitus; T2DM = type 2 diabetes mellitus; VRE = vancomycin-resistant Enterococci.

CONSORT 2010 Flow Diagram

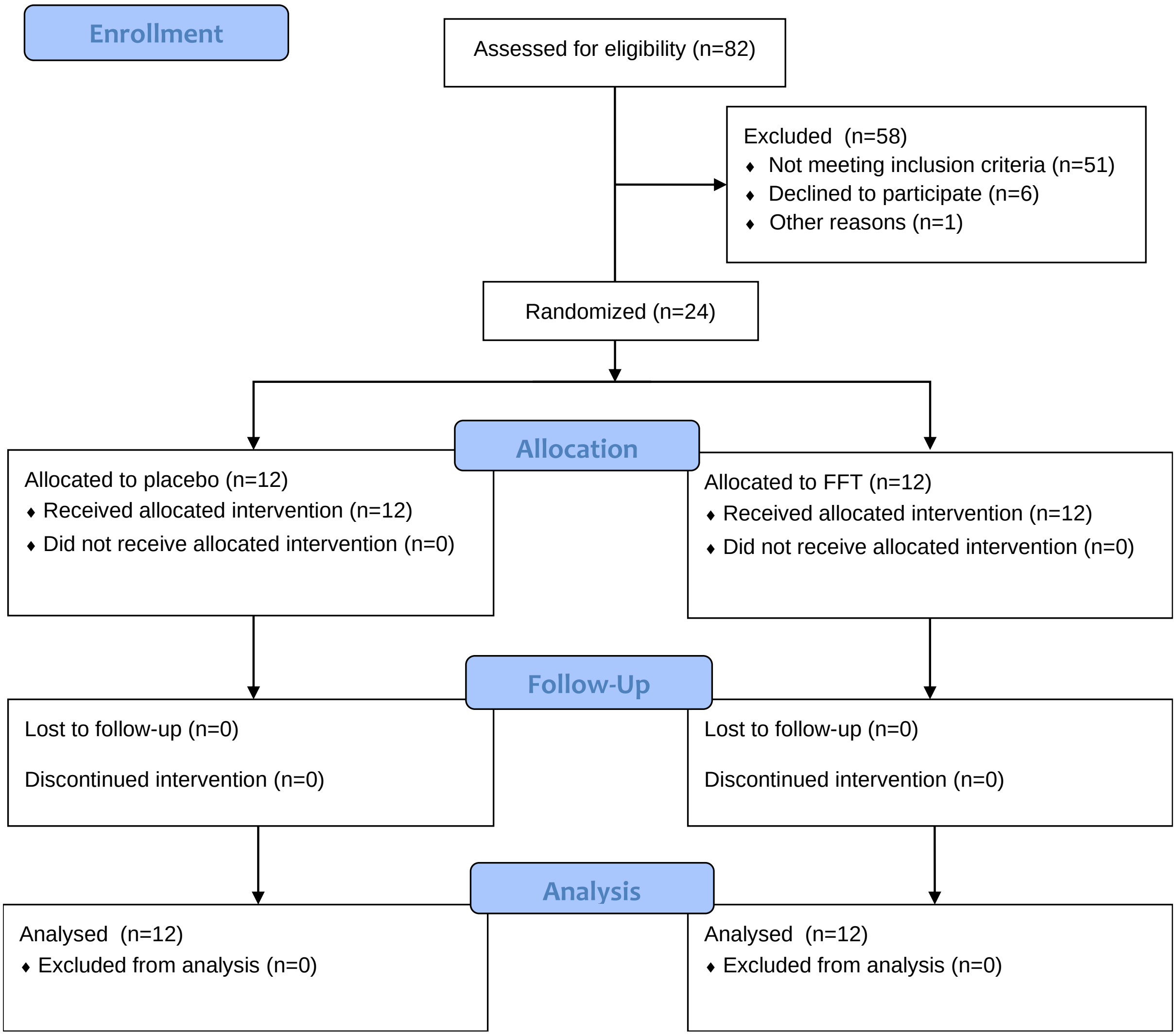

Supplement: Supplementary file 1 — Supplementary Information [file 41467_2023_41329_MOESM1_ESM.pdf]
