# Supplementary material for: Phage-microbe dynamics after sterile faecal filtrate transplantation in individuals with metabolic syndrome: a double-blind, randomised, placebo-controlled clinical trial assessing efficacy and safety
Source: Nat Commun. 2023 Sep 12;14:5600. doi: 10.1038/s41467-023-41329-z (PMC10497675; doi:10.1038/s41467-023-41329-z)
Supplement: Supplementary file 3 — Description of Additional Supplementary Files [file 41467_2023_41329_MOESM3_ESM.docx]

**Description of Additional Supplementary Files**

**File Name: Supplementary Data 1**

**Description:** Differentially abundant viral populations within the VLP phageome on day 2. Output from ANCOM-BC test.
