# Supplementary material for: Phage-microbe dynamics after sterile faecal filtrate transplantation in individuals with metabolic syndrome: a double-blind, randomised, placebo-controlled clinical trial assessing efficacy and safety
Source: Nat Commun. 2023 Sep 12;14:5600. doi: 10.1038/s41467-023-41329-z (PMC10497675; doi:10.1038/s41467-023-41329-z)
Supplement: Supplementary file 5 — Reporting Summary [file 41467_2023_41329_MOESM5_ESM.pdf]

nature portfolio

Corresponding author(s): Hilde Herrema

Last updated by author(s): Aug 18, 2023

Reporting Summary

Nature Portfolio wishes to improve the reproducibility of the work that we publish. This form provides structure for consistency and transparency in reporting. For further information on Nature Portfolio policies, see our Editorial Policies and the Editorial Policy Checklist.

Statistics

For all statistical analyses, confirm that the following items are present in the figure legend, table legend, main text, or Methods section.

n/a	Confirmed	
☐	☒	The exact sample size (*n*) for each experimental group/condition, given as a discrete number and unit of measurement
☐	☒	A statement on whether measurements were taken from distinct samples or whether the same sample was measured repeatedly
☐	☒	The statistical test(s) used AND whether they are one- or two-sided<br>*Only common tests should be described solely by name; describe more complex techniques in the Methods section.*
☐	☒	A description of all covariates tested
☐	☒	A description of any assumptions or corrections, such as tests of normality and adjustment for multiple comparisons
☐	☒	A full description of the statistical parameters including central tendency (e.g. means) or other basic estimates (e.g. regression coefficient) AND variation (e.g. standard deviation) or associated estimates of uncertainty (e.g. confidence intervals)
☐	☒	For null hypothesis testing, the test statistic (e.g. *F*, *t*, *r*) with confidence intervals, effect sizes, degrees of freedom and *P* value noted<br>*Give P values as exact values whenever suitable.*
☒	☐	For Bayesian analysis, information on the choice of priors and Markov chain Monte Carlo settings
☒	☐	For hierarchical and complex designs, identification of the appropriate level for tests and full reporting of outcomes
☒	☐	Estimates of effect sizes (e.g. Cohen's *d*, Pearson's *r*), indicating how they were calculated

Our web collection on statistics for biologists contains articles on many of the points above.

Software and code

Policy information about availability of computer code

Data collection	Data were captured with electronic case report forms build in Castor EDC (v2019.2.0-2020.2.25). Electronic medical record software from EPIC (versions August 2019-November 2020) is used in the Academic Medical Centre (AMC) Amsterdam to record results of biochemical diagnostic tests. The Eetmeter (v2019-2020) from the Voedingscentrum was used as online food diary, from which raw data was exported as Excel files. The Freestyle Libre 1 sensors and scanners were used for continuous glucose monitoring, and raw data was exported from the scanners as text files.
Data analysis	R v4.2.1, CGDA v0.8.2, fastp v0.23.2, bbmap v38.90, metaSPAdes v3.15.5, MEGAHIT v1.2.9, virsorter v2.2.3, checkv v1.0.1, BLAST v2.12.0+, vContact2 v0.11.3, bowtie2 v2.4.2, coverm filter v0.6.1, bedtools v2.27.1, samtools v1.15.1, mOTUs v3.0.3, jgi_summarize_bam_contig_depths v2.15, metabat2 v2:2.15, checkm v1.2.1, GTDB-Tk v2.1.1, CRISPCasFinder v4.2.20, vegan R package v2.6-4, MASS R package v7.3-58.1, ANCOM-BC v1.2.2

For manuscripts utilizing custom algorithms or software that are central to the research but not yet described in published literature, software must be made available to editors and reviewers. We strongly encourage code deposition in a community repository (e.g. GitHub). See the Nature Portfolio guidelines for submitting code & software for further information.

Data

Policy information about availability of data

All manuscripts must include a data availability statement. This statement should provide the following information, where applicable:

- Accession codes, unique identifiers, or web links for publicly available datasets
- A description of any restrictions on data availability
- For clinical datasets or third party data, please ensure that the statement adheres to our policy

The sequencing data generated in this study have been deposited in the European Nucleotide Archive database under accession code: PRJEB60691. The data are freely available without restriction. Source data are provided with this paper. The R207-v2 database package of GTDB-Tk is available from: https://data.gtdb.ecogenomic.org/releases/release207/207.0/auxillary_files/gtdbtk_r207_v2_data.tar.gz. Individual participant data that underlie the results reported in this article, after deidentification, are freely available in the Source Data file. The study protocol is included with the publication. All of the individual participant data collected during the trial, after deidentification, are available upon request. Methodological sound proposals with clear aims should be directed to h.j.herrema@amsterdamumc.nl. To gain access, data requestors will need to sign a data transfer agreement. Data will be available up to five years following article publication.

Research involving human participants, their data, or biological material

Policy information about studies with human participants or human data. See also policy information about sex, gender (identity/presentation), and sexual orientation and race, ethnicity and racism.

Reporting on sex and gender	All analysis pertained to people of both sexes, information on which was self-reported. Where applicable, analyses were adjusted for the effects of sex as a confounding factor. No separate sex-based analyses were performed due to the lack of statistical power.
Reporting on race, ethnicity, or other socially relevant groupings	Not applicable
Population characteristics	Recipients all suffered from metabolic syndrome, had an average age of 49.3 (Placebo) and 54.8 (Fecal filtrate), while donors where on average 32 years old. Full population characteristics are detailed in table 1.
Recruitment	Subjects were recruited through online advertisements on the public website "proefpersonen.nl", which is hosted by Link2Trial. In addition, advertisements were posted in local newspapers. Finally, subjects with the metabolic syndrome were contacted, who have participated in previous clinical studies within our department and gave permission to be contacted for new/follow-up studies. Interested participants received the written patient information leaflet and informed consent form (ICF), and were invited for a screening visit during which the study for explained and the ICF was signed. Figure S1 summarizes the patient recruitment.<br><br>Study participants were all European Dutch, overweight (body mass index (BMI) ≥ 25 kg/m2) subjects between 18 and 65 years of age and had to meet the National Cholesterol Education Program (NCEP) criteria for the metabolic syndrome. Main exclusion criteria were the use of any medication, illicit drug use, smoking, or alcohol abuse in the past 3 months, as well as a history of cardiovascular, gastrointestinal, or immunological disease. Table S4 summarises all in- and exclusion criteria.<br><br>While there was a possibility of self-selection bias, this was unlikely to affect the outcome due to the randomized, double-blinded nature of the study, and the dependence on physical measures.
Ethics oversight	Medical Research Ethics Committee Academic Medical Center Amsterdam (METC 2018_231)

Note that full information on the approval of the study protocol must also be provided in the manuscript.

Field-specific reporting

Please select the one below that is the best fit for your research. If you are not sure, read the appropriate sections before making your selection.

☒ Life sciences ☐ Behavioural & social sciences ☐ Ecological, evolutionary & environmental sciences

For a reference copy of the document with all sections, see nature.com/documents/nr-reporting-summary-flat.pdf

Life sciences study design

All studies must disclose on these points even when the disclosure is negative.

Sample size	Based on previous data from our group in which individuals with MetSyn received an FMT, and the hypothesis that a faecal phage transplant can be equally effective as a traditional FMT, we assumed a 15% improvement in glucose tolerance upon FFT. With a two-sided 5% significance level and a power of 80%, a sample size of 12 patients per group was necessary, given an anticipated dropout rate of 10%. To recruit 24 individuals with MetSyn, we anticipated a 12-month inclusion period.

Data exclusions	No data were excluded from the analyses.
Replication	Measures of blood samples (e.g., glucose, c-peptide, insulin, HbA1c) were done in triplicate. No other attempts at replication were made. The clinical study was not replicated. The interventions and study procedures were described in sufficient detail to allow replication of the study.
Randomization	After passing screening, 24 subjects with MetSyn were randomised to receive a sterile FFT from a lean healthy donor or a placebo transplant. Block randomization with stratification for age and sex was used. CASTOR EDC was used to perform the randomization, which was done by a research assistant who prepared the allocated intervention.
Blinding	Both participants and researcher were blinded for the intervention during data collecting and analysis. Both placebo and FFT interventions looked identical (clear, brown solution).

Reporting for specific materials, systems and methods

We require information from authors about some types of materials, experimental systems and methods used in many studies. Here, indicate whether each material, system or method listed is relevant to your study. If you are not sure if a list item applies to your research, read the appropriate section before selecting a response.

Materials & experimental systems

n/a	Involved in the study	
☒	☐	Antibodies
☒	☐	Eukaryotic cell lines
☒	☐	Palaeontology and archaeology
☒	☐	Animals and other organisms
☐	☒	Clinical data
☒	☐	Dual use research of concern
☒	☐	Plants

Methods

n/a	Involved in the study	
☒	☐	ChIP-seq
☒	☐	Flow cytometry
☒	☐	MRI-based neuroimaging

Clinical data

Policy information about clinical studies

All manuscripts should comply with the ICMJE guidelines for publication of clinical research and a completed CONSORT checklist must be included with all submissions.

Clinical trial registration	The study was registered at the Dutch National Trial Register (NTR) under NL8289 on the 15th of January 2020, while the first patient was included in October 2019. The delay in registration was due to a miscommunication between investigators. When this mistake came to light during the first monitor visit after the first three patients had been included, the study was directly registered at the NTR. This registry does not exist anymore and all data has been added unaltered to the Dutch Trial Register (LTR) under https://clinicaltrialregister.nl/en/trial/26916. While these data are automatically included in the International Clinical Trial Registry Platform (ICTRP), thereby fulfilling the requirement of prospective registration as required by the International Committee of Medical Journal Editors (ICMJE), it was unfortunately no longer possible to adjust the data.
Study protocol	The final version of the study protocol is included with the submission
Data collection	This was a single-center, prospective, double-blinded, randomised, placebo-controlled intervention study that was performed in our academic hospital (AMC Amsterdam). A total of 82 subjects signed the informed consent form and were screened from October 2019, of which 24 subjects were included and finished the study before December 2020.
Outcomes	The primary outcome was change in glucose metabolism, as determined by the total area under the curve (AUC) for glucose excursion during the OGTT. Secondary outcomes related to glucose metabolism were changes in fasting glucose, insulin, HOMA-IR, and HbA1c between baseline and follow-up after 28 days, as well as changes in glucose variability measured by CGM a week before and after intervention. Other secondary outcomes were the dynamic changes in gut bacteriome and virome populations following FFT or placebo intervention and the comparison of phage composition between lean donors and subjects with MetSyn. Finally, we assessed the safety of the FFT as determined by the occurrence of (serious) adverse events, physical exam, and several blood parameters for renal and liver function and inflammation. For a more detailed description of the procedures how these outcome measures were determined (e.g. the OGTT or CGM), we refer to the methods section of the manuscript.
